# Intestinal Dysbiosis in, and Enteral Bacterial Therapies for, Systemic Autoimmune Diseases

**DOI:** 10.3389/fimmu.2020.573079

**Published:** 2020-10-28

**Authors:** Eric Marietta, Ashutosh K. Mangalam, Veena Taneja, Joseph A. Murray

**Affiliations:** ^1^Department of Gastroenterology and Hepatology, Mayo Clinic Rochester, Rochester, MN, United States; ^2^Department of Immunology, Mayo Clinic Rochester, Rochester, MN, United States; ^3^Department of Dermatology, Mayo Clinic Rochester, Rochester, MN, United States; ^4^Department of Pathology, University of Iowa, Iowa City, IA, United States

**Keywords:** bacterial, monotherapies, autoimmune, microbiome, probiotic, treatment

## Abstract

Recent studies have shown that a number of common autoimmune diseases have perturbations of their intestinal microbiome (dysbiosis). These include: Celiac Disease (CeD), Multiple Sclerosis (MS), Rheumatoid Arthritis (RA), Sjogren’s Syndrome (SS), and Type 1 diabetes (T1D). All of these have intestinal microbiomes that are different from healthy controls. There have been numerous studies using animal models of single probiotics (monoclonal) or mixtures of probiotics (polyclonal) and even complete microbiota transfer (fecal microbial transfer-FMT) to inhibit or delay the onset of autoimmune diseases such as the aforementioned common ones. However, proportionally, fewer clinical trials have utilized monoclonal therapies or FMT than polyclonal therapies for treating autoimmune diseases, even though bacterial mono-therapies do inhibit the development of autoimmune diseases and/or delay the onset of autoimmune diseases in rodent models of those autoimmune diseases. In this review then, we review the previously completed and currently ongoing clinical trials that are testing bacterial therapies (FMT, monoclonal, and polyclonal) to treat common autoimmune dseases and discuss the successes in using bacterial monotherapies to treat rodent models of these common autoimmune diseases.

## Introduction

The definition of autoimmune disease first arose with Dr. Paul Ehrlich, wherein he described the condition as “horror intoxicus” (1). Currently Medline Plus.gov has the definintion of autoimmunity as “when the body’s immune system attacks and destroys healthy body tissue by mistake” (https://medlineplus.gov/ency/article/000816.htm), and the website for the American Autoimmune Related Diseases Association (AARDA) has over 100 diseases listed as being autoimmune (https://www.aarda.org/diseaselist/). Many studies have been done where probiotics have been given to animal models of the less common autoimmune diseases. The most common autoimmune diseases as listed by Medline Plus.gov are: Addison disease, Celiac Disease, dermatomyositis, Graves disease, Hashimoto thyroiditis, multiple sclerosis, Myasthenia gravis, Pernicious anemia, Reactive arthritis, Rheumatoid arthritis, Sjogren syndrome, Systemic lupus erythematosus, and type 1 diabetes. This review will focus on these common autoimmune diseases, and more specifically, those autoimmune diseases that have had clinical trials conducted (or are being conducted) to treat autoimmune patients with bacterial therapies (fecal microbial transplantation and probiotic) as listed by clinicaltrials.gov. This therefore excludes Addison disease, dermatomyositis, Graves disease, Hashimoto Thyroiditis, Myasthenia gravis, Pernicious anemia, and Systemic Lupus Erythematosus.

The composition of the intestinal microbiome of many autoimmune diseases, including celiac disease (CeD), multiple sclerosis (MS), rheumatoid arthritis (RA), Sjogren‘s syndrome (SS), and type 1 diabetes (T1D) has been characterized predominantly using 16s rDNA sequencing of stool samples. Studies have demonstrated that alterations of the fecal intestinal (colonic) microbiome (dysbiosis) exist in patients that have CeD, MS, RA, SS, and/or T1D, and that MS patients may have a uniform decrease in *Prevotella* (2–14).

One study done with T1D patients demonstrated that there was also dysbiosis in the small bowel of T1D patients as compared to controls (15). Additionally, a study with duodenal biospises of MS patients found dysbiosis in their small intestine as well (16). In addition, a number of studies found dysbiosis in the small bowel of CeD patients (6, 7, 17–21). All of these studies clearly demonstrate that patients with these autoimmune diseases have dysbiosis in their colonic microbiomes, where the composition of their fecal microbiome is different from controls such as first degree relatives or healthy controls. The data for dysbiosis in the small intestine of CeD is strong, but more research needs to be done with MS, RA, SS, and T1D.

It has been often assumed microbial therapies work by normalizing the resident microbiota, and hence, prevent or treat autoimmune diseases that have associated dysbiosis. How the change in the composition of the intestinal microbiome due to microbial therapy would exert changes in the systemic immune system has been a focus of many rodent model studies. At least two pathways have been identified. The first is through Pattern Recognition Receptors (PRRs), such as Toll-Like Receptors (TLRs), on different cell types that interact with bacteria in the lumen, and the PRRs would detect and bind to Microbe Associated Molecular Patterns (MAMPs) expressed by the bacteria in the intestine (22, 23). Rodent models for MS have demonstrated a role for TLR2 in controlling and treating disease (24, 25).

The second pathway would be through the production of Short Chain Fatty Acids (SCFAs) by the bacteria. The SCFAs would bind to SCFA receptors expressed by the responsive host immune cell, resulting in a phenotypic change to being either regulatory or inflammatory, and deficits in SCFA production have been identified in multiple sclerosis (5, 26, 27).

However, normality for the human intestinal microbiome is unclear and varies greatly by geography, diet, and other external factors (28–31) . Even age affects the composition of the human intestinal microbiome, such that there are at least four distinct age groups in which the human intestinal microbiome is different (infant, pre-adolescent, adult, elderly) (32–35). Genetics play a crucial role as well, and rodent models have demonstrated that even the smallest alterations in the genetic background can lead to changes in the composition of the intestinal microbiome (36–39). Differences in the composition of the intestinal microbiome that are due to the effect of age, diet, geography, and host genetics, could potentially also contribute to different responses to different bacterial therapies, although the specific differences as a consequence of these factors, especially age and genetics, in response to bacterial therapies have not been rigorously addressed, especially in humans.

**Figure 1:**
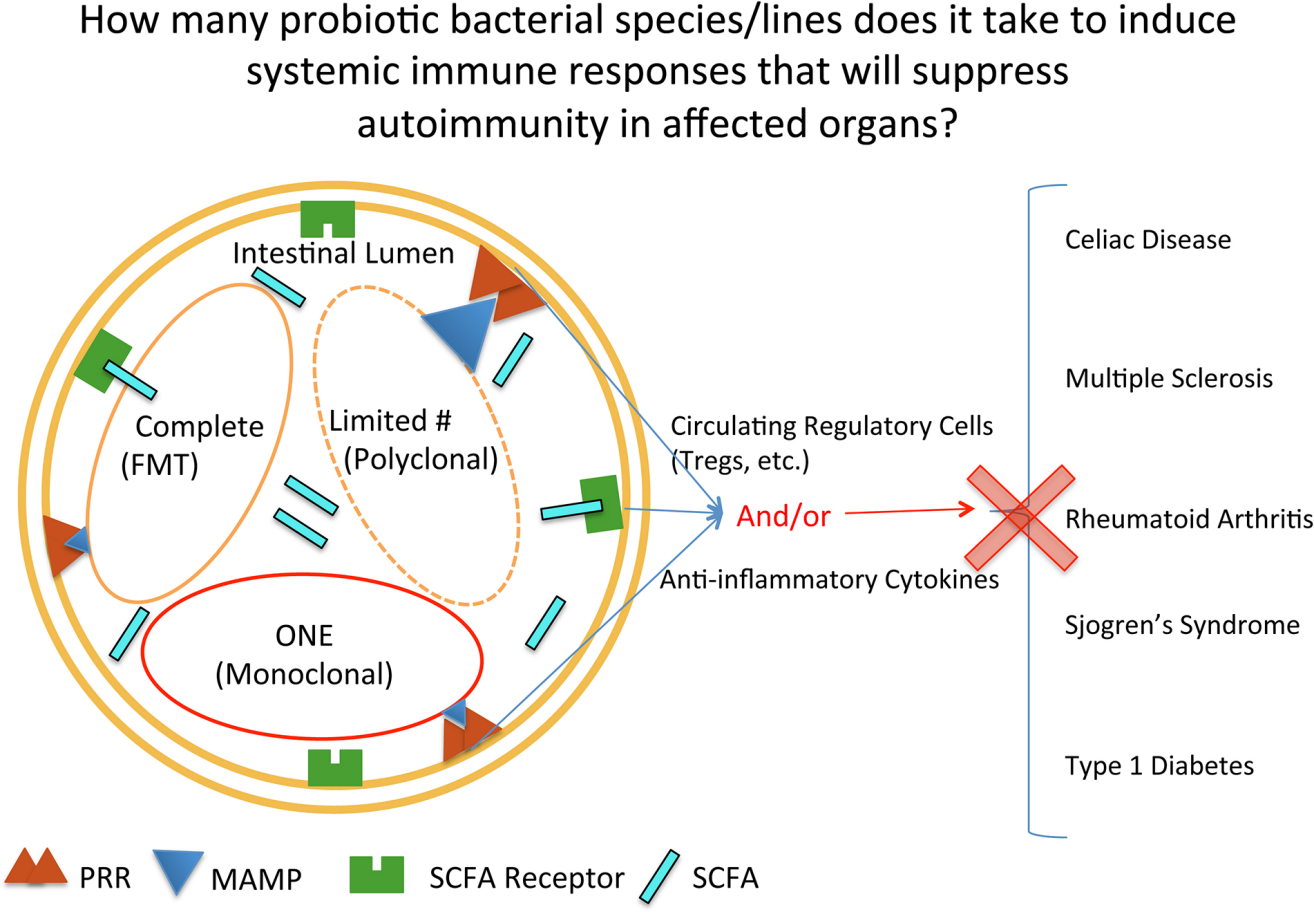
Mechanisms by which probiotic bacterial therapies can affect inflammation at intestinal and extraintestinal sites. Depicted in the intestinal lumen are the three main categories of bacterial groups currently used in clinical trials, reflecting quantity: complete, restricted mix (Limited#/Polyclonal), and one (monoclonal). PRR, pattern recognition receptor; MAMP, microbe associated molecular pattern; SCFA, short chain fatty acid; SCFAR, SCFA Receptor.

Despite the complexity of the effects of probiotic treatment, the concept of probiotics as being beneficial in helping the intestine stabilize bacterial content (reach homeostasis) is currently well known publically. The concept of how much bacteria and which types of bacteria are needed to achieve homeostasis is not as well known publically, nor determined in a truly rigorous scientific manner (**Figure 1**). The transfer of complete microbial content, or Fecal Microbial Transplantation (FMT), interestingly was done as long ago as the fourth century in China to treat diarrhea, constipation, and abdominal pain (40). In this type of treatment today, all of the fecal bacteria is transferred. Currently, the greatest success story of FMT is with the treatment of patients infected with *Clostridium difficile* (*C. difficile*) (41, 42). Mixes of probiotics, such as in the form of yogurt, are not a complete mix of the bacteria found in the digestive tract of humans. And at the opposite spectrum, there are many studies done with rodents where only one bacteria is provided to the animal. With autoimmune diseases, FMTs and probiotics (both mixes and single strains) are being used in clinical trials for treating patients with autoimmune disease. In addition, there is a fourth category, that of bioengineered probiotics/bacteria that secrete proteins to reduce autoimmune responses. This will be highlighted in the last section on type 1 diabetes, as there is currently an ongoing clinical trial testing such a product. In order therefore to obtain insight into the effectiveness of the three main types of intestinal microbial treatments (complete, restricted mix, and monoclonal) used in treating autoimmune diseases, this review will systematically progress through each of the common autoimmune diseases that have had clinical trials conducted where probiotics (including FMTs) were given to autoimmune patients to treat their disease (CeD, MS, RA, SS, and T1D).

## Autoimmune Diseases

### Celiac Disease

There is currently one clinical trial (NCT 04014413) that is recruiting Celiac patients for conducting FMT. This study is also determining the efficacy of FMT for a number of other autoimmune and inflammatory disorders and is still recruiting patients. No data has been released yet. In contrast, there have been many randomized, double blind, placebo clinical trials testing monoclonal and polyclonal bacterial therapies with Celiac Disease that have been completed and results published, with at least four (43–46) that are monoclonal and three polyclonal (47–49), with the earliest results published as far back as 2013 (46). Bacteria used in the bacterial monotherapies were *Bifidobacterium infantis* (45, 46), *Bifidobacterium breve* (43), and *Bifidobacterium longum* (44). The rest were mixes of two or more bacterial strains (47–49). In the clinical trial with *B. breve* (43), circulating levels of TNFα present in children on a GFD were decreased with the administration of *B. breve*. This clinical trial was based in part on the following mouse models (50, 51), wherein *B. breve* induced regulatory T cells (Tregs) in mice. With the administration of *B. longum* to children with CeD (44) there was a significant reduction in the number of circulating CD3+ T lymphocytes and a slight reduction in the level of TNFα. This was based in large part on a previous study with a rat model of enteropathy, wherein administration of *B. longum* to the rats increased IL10 and decreased TNFα (52). With the administration of *B. infantis* to adults (46), there was an improvement in the gastrointestinal rating scale for indigestion, constipation, and reflux, and a later publication (45) found that *B. longum* affected the innate immune system of patients by decreasing α-defensin 5. Previous studies had shown that the administration of *B. infantis* to mouse models of colitis and bacterial inflammation (53–55) had increased the number of Tregs and downregulated inflammatory cytokines. In the publication on a polyclonal treatment by Francavilla et al. (47), five strains of lactic acid bacteria and *bifidobacteria* were given to adult Celiac patients with IBS like symptoms. The administration of the probiotic mix led to a significant decrease in the gastrointestinal symptom rating scale, whereas the placebo did not. In the publication on the administration of two *lactobacillus* strains (*L. plantarum* HEAL9 and *L. paracasei* 8700:2) to children who were tTG IgA positive but still on a gluten containing diet, a detailed analysis of circulating lymphocytes was done by flow cytometry (48). The most notable change was a significantly reduced expansion of CD4+CD25+CD45RO+ T cells (effector and memory T cells) in the probiotic group (48). No significant change was observed in the levels of circulating anti tTG IgA levels (48). With the third clinical trial that administered a mix of probiotic bacteria, VSL#3 was administered to adult CeD patients who still had gastrointestinal symptoms, despite being on a gluten free diet (49). CeD patients who received the VSL#3 probiotic mix had no improvement in clinical gastrointestinal symptoms over the placebo group (49). The CeD patients also did not have significant changes to their gut microbiome as measured by microscopic and molecular analysis (49).

### Multiple Sclerosis

Three clinical trials have been set up for conducting FMT with MS patients (NCT03183869), (NCT03975413), and (NCT04150549). Only 10 patients were recruited for the NCT03183869 study due to early termination (the primary investigator passed away), and no significant changes in circulating cytokines were observed (either inflammatory or anti-inflammatory) in the small number of patients. NCT04150549 has not started recruiting patients, and NCT03975413 is still active. Rodent models of FMT to treat EAE provide mixed results. One study with rats show that transfer of fecal microbiota of EAE resistant rats (Albino Oxford) to EAE rats ameliorates the disease in disease prone rats (Dark Agouti) (56). In contrast, another study observed that the transfer of fecal microbota from naïve mice to EAE mice did not ameliorate disease (37). It was only when fecal microbiota was transferred from CD44 knock out mice that disease was ameliorated (37).

No clinical trials with bacterial monotherapies have been conducted for MS patients. However, in one randomized, double blind, placebo-controlled study conducted at the Islamic Azad Medical Center, capsules containing probiotic bacteria, *Lactobacillus acidophilus, Lactobacillus casei, Bifidobacterium bifidum* and *Lactobacillus fermentum*, were administered to relapsing remitting MS patients (RRMS) (57). Patients who received the probiotic mix had significantly decreased EDSS (expanded disability status scale) and DASS (depression, anxiety, and stress scale). Another study conducted at Harvard Medical Center administered a very common and commercially available probiotic mix, called Visbiome, which was previously called VSL#3, to MS patients and healthy controls (58). Since no placebo was tested, this study was not a randomized double blind placebo trial. The mix contained 8 different strains of bacteria: *Lactobacillus paracasei* DSM 24734, *Lactobacillus plantarum* DSM 24730, *Lactobacillus acidophilus* DSM 24735, and *Lactobacillus delbruckeii* subspecies *bulgaricus* DSM 24734), *Bifidobacterium longum* DSM 24736, *Bifidobacteriuminfantis* DSM 24737, and *Bifidobacterium breve* DSM24732), and *Streptococcus thermophilus* DSM 24731). RRMS patients who received Visbiome had changes to their gut microbiome composition, and healthy controls who received Visbiome had a decreased alpha diversity.

### Rheumatoid Arthritis

One clinical trial (NCT03944096) is currently being conducted with testing FMT for treating RA patients. Its title is Efficacy and Safety of Fecal Microbiota Transplantation in Patients With Rheumatoid Arthritis Refractory to Methotrexate (FARM). No results have been posted yet though.

Only one group, at the Tehran University of Medical Sciences, has published results on providing a bacterial monotherapy for treating RA. They did a randomized, double- blind, placebo-controlled clinical trial using capsules that contained active *L. casei* (59). They obtained similar results with the women with RA, in that inflammatory cytokines went down and anti-inflammatory IL-10 went up. The disease scores for tender and swollen joints for the patients that received the *L. casei* were also decreased as compared to the placebo. *L. casei* was tested previously for its effectiveness in mouse and rat models of arthritis to decrease the inflammation of arthritis in those models (60–62). These studies showed that in the animal models, the administration of *L. casei* resulted in decreased incidence and development of arthritis that was associated with decreased production of inflammatory cytokines such as IFNγ, IL-17, and TNFα, and a decrease in the production of anti collagen antibodies. A different randomized, double- blind, placebo-controlled clinical trial conducted at the Kashan University of Medical Sciences, RA patients were given *L. casei* in addition to *Lactobacillus acidophilus* and *Bifidobacterium bifidum* in a capsule. The disease activity score of 28 joints was decreased significantly in the probiotic treated group; however, the tender and swollen joints scores individually were not decreased (63). A second group gave *Lactobacillus rhamnosus* GR-1 and *Lactobacillus reuteri* RC-14 in capsules to RA patients in a randomized, double- blind, placebo-controlled clinical trial conducted at the St. Joseph’s Health Care in London, Ontario, Canada (64). They did not observe an overall clinical improvement in the probiotic arm, but they did find that blood levels of GMCSF, MIP1α, TNFα, IL-6, IL12p70, IL-15, and IL-17, as determined by multiplex immunoassay, were decreased in the probiotic arm.

Thus, although there is only one published clinical trial for administering a bacterial monotherapy to RA patients, the results do suggest that such therapies can be effective in reducing inflammation in RA patients and that this is associated with increased systemic production in anti inflammatory cytokines such as IL10 and decreased production of inflammatory cytokines.

### Sjogren’s Syndrome

One clinical trial for treating Sjogren’s Syndrome with FMT has been completed (NCT03926286). No results have been posted yet though. Results in rodent models of fecal microbiota transfer to treat SS has been promising though. In one study, the transfer of fecal microbiota of disease-free mice to a mouse model of SS did ameliorate the disease (65). A second study using a different mouse model of SS had similar results in that transfer of fecal microbiota from disease free mice reverted the disease along with a decrease in the pathogenic CD4+IFNγ+ T cells (66).

No clinical trials for SS using a monoclonal bacterial therapy have been established yet. However, there is a clinical trial set up to determine the efficacy of treating oral candidiasis in SS (NCT03840538) with probiotics, and this has been completed and results published (67). Here a mix of probiotics was given (*Lactobacillus acidophilus*, *Lactobacillus bulgaricus*, *Streptococcus thermophilus* and *Bifidobacterium bifidum*) to SS patients to determine if that would decrease candidial load. Results from the study showed that this mix did provide a statistically significant decrease in the candidial load from baseline to treatment end, and the difference was not statistically significant in the placebo group. There is one publication that showed that administering a probiotic mix to a mouse model of dry eye, the main symptom of primary SS, does have a beneficial effect (68). In this study, a mix called IRT5 was used (*Bifidobacterium bifidum*, *Lactobacillus acidophilus*, *Lacto- bacillus casei*, *Lactobacillus reuteri*, and *Streptococcus thermophilus)* (68). The administration of these bacteria orally by gavage led to increased levels of CD11c+ and CD11b+ cells in the spleen and increased levels of IL-10 along with decreased levels of IL1β in the conjuctiva and cornea of the eye (68).

### Type I Diabetes

Currently there is one clinical trial designed to determine the benefit of FMT for treating T1D (NCT04124211). This is still recruiting patients, so no results have been posted.

With T1D, only one bacterial monotherapy placebo-controlled, double blind, randomized clinical trial has been completed and results published (69). In that study, conducted at the University Medical Center in Ahvaz, Iran, patients were administered a synbiotic mixture of *Lactobacillus sporogenes* and a corresponding prebiotic, fructooligosaccharide for 8 weeks to children diagnosed with T1D (69). The synbiotic mix improved the following glycemic indices in the children as compared to a placebo control group: FBG, HbA1c, insulin, hs-CRP, and TAC. This was preceded by a study in an animal model in which *Bifidobactera bifidum* and *Lactobacillus sporogenes* were administered separately to rats that developed paw edema due to injection with carrageenan (70). Administration of either bacterial strain alone to the rats resulted in decreased paw thickness, and increased physical activity as determined by a stair climbing assay and motility assay (70). Another clinical trial is set up to administer a bacterial monotherapy (NCT03961347), but it is still recruiting patients at the University of Florida. In this clinical trial, *L. johnsonii* is being administered to adults with T1D. This clinical trial was preceded by studies in animal models as well (71–76). Three other clinical trials have been set up to test the effect of polymicrobial therapies on T1D, but no publications on the results have yet emerged (NCT03032354 (77), NCT03880760, and NCT03423589). The first multiple bacterial therapy clinical trial (NCT03032354) was set up by Groele et al. to be conducted at the Medical University of Warsaw, Poland and the Department of Endocrinology and Diabetology, Children’s Memorial Health Institute in Warsaw, Poland (77). Children with T1D were to be recruited and given a mix of *L. rhamnosus* and *B. lactis* Bb12. The second one with multiple bacteria (NCT03880760), was set up to be conducted at the China Medical University Hospital in Taichung, Taiwan. It was designed to treat T1D children with a mix *of Lactobacillus salivarius + Lactobacillus johnsonii + Bifidobacterium lactis*. The third multiple bacterial therapy clinical trial, NCT03423589, was designed to administer to T1D patients VSL#3 (Visbiome) at the Medical School of Wisconsin. As described previously, VSL#3 is a mix of 8 bacterial strains. Overall then, only one clinical trial has been completed and published on the efficacy of administering bacteria to treat T1D, and this used a bacterial monotherapy and had promising data.

NCT03751007 is a clinical trial that is utilizing bacteria from the fourth category, genetically engineered bacterial, *Lactococcus lactis* that secretes proinsulin and IL10 (AG019-Precigen Actobio T1D, LLC), and is currently (as of this publication) being conducted at 18 sites in the United States and Belgium. This genetically modified bacterial strain was tested first in NOD mice (76). Administration of the *L. lactis* secreting proinsulin and IL10 along with anti CD3, substantially decreased hyperglycemia in the NOD mice (76).

## Conclusions/Discussion

Of the five common autoimmune diseases that had clinical trials to test bacterial therapies (CeD, MS, RA, SS, and T1D), all five had FMT being tested. One for Celiac disease, three for MS, one for RA, one for SS, and one for T1D (7 in total). Only one had truly completed (NCT03926286), which is the one determining the efficacy of treating SS with FMT. No results from that clinical trial have been posted yet. Another one (NCT03183869) for MS had terminated early, but had posted results on circulating cytokines. Thus, it is too early to make any predictions on the efficacy of treating autoimmune diseases with FMT; however, there is a lot of effort going into answering this question.

There were 3 clinical trials that use a restricted number of probiotic bacteria for Celiac Disease, 2 for MS, 3 for RA, 1 for SS, and 3 for T1D (12 in total). This is about twice as many as the number of clinical trials to evaluate the efficacy of treating autoimmune diseases with FMT. These gave mixed results in that some studies showed improvement in gastrointestinal symptoms, but others did not. Also too, not all of the studies evaluated immune responses in a similar way, making it difficult to determine if there was a common response by the immune system to these mixes of probiotic bacteria, most especially in those trials where no beneficial changes were observed to occur in gastrointestinal symptoms.

For the administration of monoclonal therapies to patients with autoimmune diseases, there were 4 with celiac disease, 0 for MS, 1 for RA, 0 for SS, and 2 for T1D (7 total). Results from the Celiac monoclonal clinical trials had results that showed that administrating monoclonal therapies decreased levels of sera TNFα and circulating CD3+ T cells. Also too α-defensin5 of the innate immune system was decreased. In addition, gastrointestinal symptoms improved with the administration of monoclonal bacterial therapy in CeD patients. Similarly in RA patients, using only *L. casei*, inflammatory cytokines went down and anti-inflammatory IL10 went up. Symptoms and disease scores improved as well. With the one monoclonal therapy on T1D that was published, glycemic indices were improved with the administration of *L sporogenes*. Overall then, there is a deficit in the data on the outcomes of monoclonal bacterial therapies for treating autoimmune patients, but the limited data suggests that, at least with CeD and RA, there is a decrease in systemic inflammatory cytokines that is associated with an increase in anti inflammatory cytokines. The rodent models of these diseases and the administration of these same monoclonal bacteria support these findings as well as suggest that regulatory T cells are increased by the administration of these bacteria. It should be noted though, that the rodent models of autoimmune diseases treated with the probiotic mixes had similar results as well.

In addition, there are many other rodent models of monoclonal therapies to treat these common autoimmune diseases, all of which have similar findings in that regulatory T cells are increased along with anti inflammatory cytokines and other regulatory cells. One example is with *Prevotella histicola* in a CIA model, in which *P. histicola* induced IL-10, an anti-inflammatory cytokine, in the intestines (jejunum) of the treated mice (78). Serum levels of IL10 also increased after two weeks of administering *P. histicola*, as well as levels of regulatory T cells and a corresponding decrease in levels of Th17 cells (78). With the EAE model, *P. histicola* induced the production of IL10 in dendritic cells and macrophages and increased levels of regulatory T cells (79). Both of these studies demonstrate that *P. histicola* has anti-inflammatory properties that lead to the generation of regulatory T cells. With T1D, there have been a couple of studies that have shown that bacterial monotherapies work to decrease the incidence of T1D in NOD mice by increasing the levels of regulatory T cells (80, 81). In the one study with *Clostridium*, the Tregs would be generated in the intestine and then migrate to the pancreatic lymph nodes (80). And with the study with *Akkermansia muciniphilia*, increased numbers of Tregs generated by *A. muciniphilia* were associated with increased levels of IL10 and TGFβ (81). Other rodent studies that have monoclonal therapies for Celiac Disease include treatment with *P. histicola* (82), *B. longum* (52, 83, 84), *L. rhamnosus* (85) and *L. casei* (86) For MS, there are rodent EAE models using *Lactobacillus reuteri* (87) and *Lactobacillus plantarum* (88). For RA, there are rodent models using *L. casei* (60, 62, 89), *Bifidobacterium animalis* (90), *Lactobacillus fermentum* (91), *L. plantarum* (92), *L. rhamnosus* (93, 94). For T1D, there is another rodent model that uses a monoclonal therapy, but a different species of *Lactobacillus*, *Lactobacillus brevis* (95). Thus, there is a large number of probiotic monotherapies for autoimmune diseases that have been tested in rodent models, but relatively few have been incorporated into clinical trials.

As to the fourth category of probiotics, that of bioengineered bacteria that secrete specific disease associated antigens in order to suppress disease activity, there are three rodent models. There is the previously mentioned study for T1D (76) that used *Lactococcus lactis* that secreted proinsulin and IL10. There is also one for a mouse model of CeD in which *Lactococcus lactis* that secreted a gliadin epitope was used (96). The third is a study that generated *lactobaccili* to express EAE antigens and then administered that to a rat EAE model (97). All three of these models using recombinant bacteria showed a decrease in disease activity with the administration of the recombinant bacteria to the rodent.

In summary, there are almost twice as many clinical trials that are being, or have been conducted, that use probiotic mixes as there are that are using monoclonal bacterial therapies or FMTs. So far, there are few rodent models of FMTs to treat the common autoimmune diseases, and the results are mixed. FMT to treat SS in rodents has provided positive results, but FMT to treat EAE in rodents has provided contrasting results. In contrast, there is a large number of rodent models of autoimmunity that demonstrate that monoclonal bacterial therapies are effective in altering the systemic immune respone in autoimmunity. Many more clinical trials should be undertaken to conclusively determine the efficacy of monoclonal bacterial therapies in treating autoimmune diseases, especially with bacterial strains that have been tested in rodent models but not yet tested in clinical trials. Since FMTs are designed to transfer all of the microbial content to the recipient and there are a number of currently ongoing clinical trials, there should be enough data in the next five years to determine the efficacy of FMTs in treating autoimmunity.

## Author Contributions

All authors contributed to the article and approved the submitted version.

## Funding

Funding is supported by grants from NIH (NIH- R01AI075262), Mayo Clinic, and the Department of Defense (DOD- W81XWH-10-1-0257).

## Conflict of Interest

The authors are all co-inventors on US patent #10,555,975 B2, entitled: Prevotella Histicola Preparations and the Treatment of Autoimmune Conditions. VT received funding from Evelo Biosciences, Inc.
